# Double Trouble in the Abdomen: Waugh Syndrome Presenting as Left-Sided Ileocolic Intussusception in an Infant

**DOI:** 10.7759/cureus.108597

**Published:** 2026-05-10

**Authors:** Hema Mohan, Minmini Selvam, Venkata Sai

**Affiliations:** 1 Radiology, Sri Ramachandra Institute of Higher Education and Research, Chennai, IND; 2 Radiology, Addenbrooke’s Hospital, University of Cambridge, Cambridge, GBR

**Keywords:** bowel ischemia, intestinal obstruction, intussusception, midgut malrotation, waugh syndrome

## Abstract

Waugh syndrome is a rare condition characterized by the coexistence of intestinal malrotation and intussusception. We report the case of a six-month-old infant presenting with rectal bleeding, non-projectile bilious vomiting, and fever. Clinical examination revealed a palpable abdominal mass on the left side of the abdomen. Imaging demonstrated ileocolic intussusception with features suggestive of underlying malrotation. During surgery, intestinal malrotation with the cecum located in the left upper quadrant and a long-segment ileocolic intussusception with gangrene were identified. The patient underwent manual reduction of intussusception, bowel resection with anastomosis, and Ladd’s procedure. Histopathology confirmed gangrenous changes in the resected bowel. The patient recovered well postoperatively and was tolerating feeds at discharge. This case highlights the importance of recognizing atypical presentations of intussusception and considering underlying malrotation, as management differs from isolated intussusception.

## Introduction

Intussusception is one of the most common causes of intestinal obstruction in infants and young children and represents a frequent pediatric surgical emergency [1]. It commonly affects children younger than two years, with peak incidence during infancy [2]. Telescoping of a proximal bowel loop into a distal segment may cause venous congestion, mural edema, and ischemia if untreated [3]. Clinical manifestations include intermittent abdominal pain, vomiting, and rectal bleeding [4].

Intestinal malrotation is a congenital anomaly caused by abnormal rotation and fixation of the primitive midgut during embryological development [5]. Disorders of rotation may result in abnormal bowel position and narrow mesenteric attachment [6]. Delayed diagnosis may lead to volvulus, obstruction, and increased morbidity [7].

The coexistence of intussusception and malrotation is termed Waugh syndrome [8]. This rare association has been described in the literature, with fewer than 100 cases reported to date, predominantly as isolated case reports and small case series [9]. Additional reports suggest the condition may be under-recognized [10].

Ultrasonography is the preferred first-line modality for diagnosing intussusception and demonstrates the classic target sign [11]. Imaging evaluation for malrotation includes assessment of bowel orientation and mesenteric vessel relationship [12]. Characteristic radiographic patterns of malrotation have also been described on cross-sectional and contrast studies [13].

When malrotation is confirmed, operative treatment generally includes reduction of intussusception followed by a Ladd procedure, which involves division of Ladd’s bands, widening of the mesenteric base, and repositioning of the bowel to reduce the risk of volvulus [14].

## Case presentation

A six-month-old male infant presented to the emergency department with a two-day history of multiple episodes of bleeding per rectum, non-projectile bilious vomiting, and fever. The child had a history of complex congenital heart disease, including atrial septal defect, ventricular septal defect, and patent ductus arteriosus, for which intracardiac repair had been performed at one month of age. On examination, the child was irritable and mildly dehydrated. Vital signs revealed a heart rate of 148 beats/minute, respiratory rate of 36 breaths/minute, temperature of 100.8°F, blood pressure of 88/54 mmHg, and oxygen saturation of 98% on room air. A palpable mass was noted on the left side of the abdomen, and digital rectal examination revealed a mass at the fingertip. Laboratory investigations showed hemoglobin of 10.8 g/dL, total leukocyte count of 15,200/mm³, and platelet count of 3.2 × 10⁵/mm³. Serum electrolytes were within normal limits, with sodium of 136 mEq/L and potassium of 3.8 mEq/L, and C-reactive protein was 12 mg/L.

**Figure 1:**
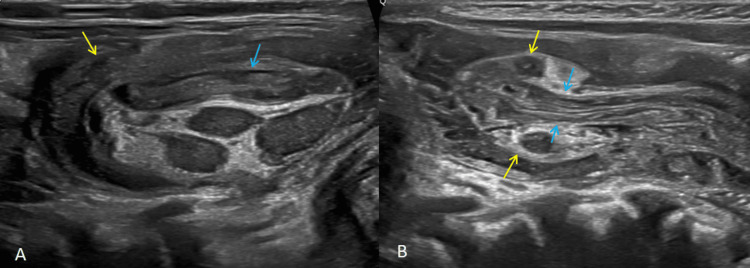
Ultrasound of the abdomen demonstrating ileocolic intussusception, showing telescoping of bowel loops with the characteristic “target” appearance. The yellow arrow highlights intussuscipiens, and the blue arrow highlights intussusceptum.

Ultrasound of the abdomen demonstrated ileocolic intussusception in the left upper abdomen, characterized by the classical “target” sign with concentric bowel loops (Figure 1).

**Figure 2:**
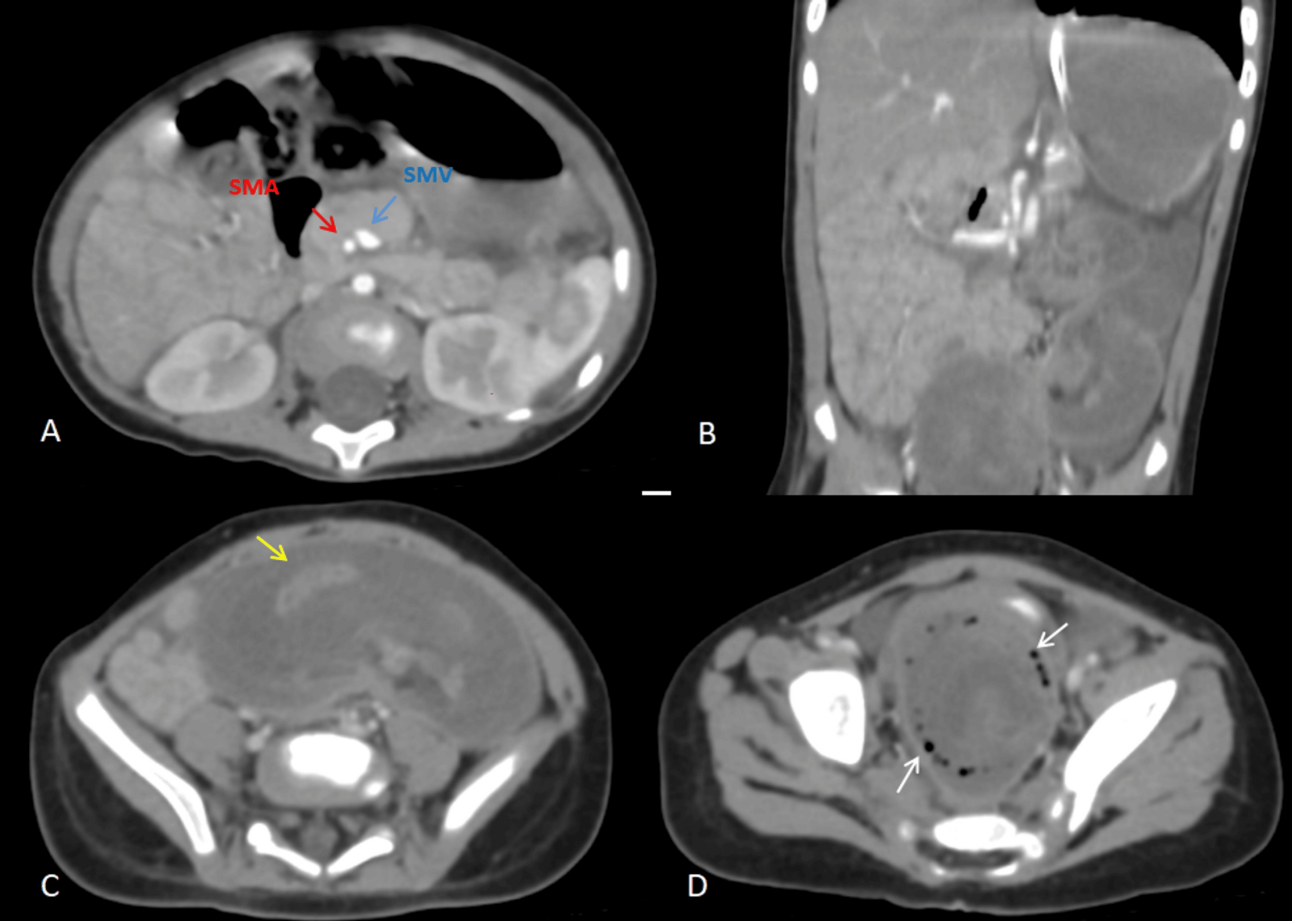
Contrast-enhanced CT of the abdomen. (A) The superior mesenteric vein is seen to the left of the superior mesenteric artery, indicating an abnormal superior mesenteric artery-superior mesenteric vein relationship. (B) Small bowel loops are predominantly on the right side, with large bowel loops on the left of the midline, consistent with intestinal malrotation. (C) Collapsed ileal loops with mesenteric fat and vessels telescoping into the lumen of the large bowel, representing ileocolic intussusception on the left side. (D) Thickened bowel loops with intramural air foci, suggestive of gangrenous changes. The red arrow depicts the superior mesenteric artery, the blue arrow depicts the superior mesenteric vein, the yellow arrow highlights Intussusceptum, and the white arrow highlights pneumatosis.

Contrast-enhanced CT of the abdomen revealed small bowel loops predominantly on the right side of the abdomen and large bowel loops predominantly on the left of the midline, consistent with intestinal malrotation. There was an inversion of the superior mesenteric artery-superior mesenteric vein axis. A long-segment ileocolic intussusception was identified in the left abdomen, with mesenteric fat and vessels telescoping into the lumen of the colon. Associated bowel wall thickening with intramural air foci suggested gangrenous changes, and mild ascites was also present (Figure 2).

**Figure 3:**
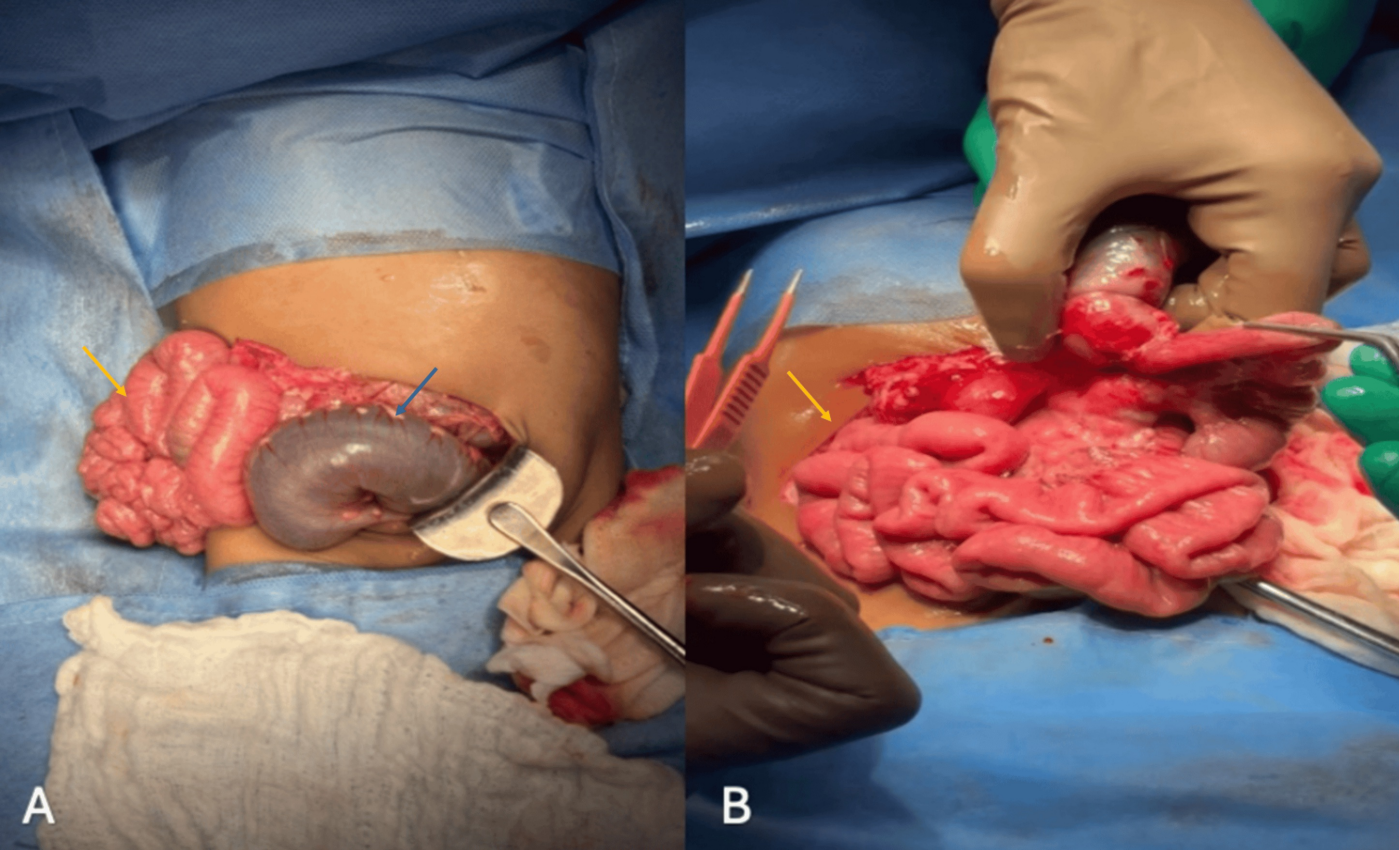
Intraoperative images demonstrating intestinal malrotation with the small bowel loops on the right side and large bowel loops on the left side with associated ileocolic intussusception and gangrenous changes of the involved bowel segment. The yellow arrow highlights small bowel loops on the right side, and the blue arrow highlights intussusception with gangrenous changes.

In view of the atypical left-sided location of intussusception, presence of bilious vomiting, and suspicion of underlying malrotation, non-operative reduction (hydrostatic or pneumatic) was not attempted, and the patient was taken up for surgical management. The patient underwent emergency exploratory laparotomy, which confirmed intestinal malrotation and a long-segment ileocolic intussusception with gangrene of the involved segment. Manual reduction was performed, followed by resection of approximately 20 cm of non-viable ileocolic bowel with primary end-to-end ileocolic anastomosis. A Ladd’s procedure, including division of Ladd’s bands, widening of the mesenteric base, and repositioning of the bowel, was also performed (Figure 3).

Postoperatively, the patient required intensive care management for five days. Broad-spectrum intravenous antibiotics (piperacillin-tazobactam and metronidazole) were administered. The postoperative course was complicated by supraventricular tachycardia on postoperative day two, which was managed with adenosine, resulting in stabilization. The patient developed hypokalemia, which was corrected with intravenous potassium supplementation. The patient was gradually weaned off oxygen support over three days. Serial lactate levels showed normalization, decreasing from 2.8 mmol/L preoperatively to 1.4 mmol/L postoperatively, indicating resolution of tissue hypoperfusion. Enteral feeding was initiated on postoperative day four following a return of bowel function and was well tolerated. The patient improved clinically and was discharged in stable condition after a total hospital stay of 10 days, including five days in the intensive care unit.

## Discussion

Waugh syndrome remains an uncommon but clinically important diagnosis [8-10]. Many children initially present with symptoms similar to isolated intussusception, including pain, vomiting, and bleeding per rectum [1-4]. However, recurrent episodes, failed enema reduction, bilious vomiting, or atypical left-sided location should raise suspicion for associated malrotation [5-7].

The proposed mechanism involves incomplete fixation of the cecum and ascending colon, producing excessive bowel mobility that predisposes the ileocecal segment to telescoping [8-10]. Careful ultrasonographic review and complementary imaging can help identify associated rotational anomalies [11-13].

Recognition of this entity changes management because correction of malrotation is required to reduce the future risk of volvulus [14]. Early diagnosis and timely surgery are associated with favorable outcomes.

## Conclusions

This case reinforces the importance of considering underlying malrotation in atypical presentations of intussusception, particularly when imaging demonstrates unusual location or abnormal mesenteric vessel orientation. Early recognition is essential to guide appropriate surgical management and prevent serious complications such as bowel gangrene and volvulus. In such cases, a lower threshold for surgical intervention should be maintained, especially when there are signs of bowel ischemia or when anatomical abnormalities are suspected, as non-operative reduction alone may be inadequate.
